# An innovative and collaborative teaching approach to delivering mental health capabilities in the UK Foundation Programme Curriculum 2021

**DOI:** 10.1192/bjb.2022.85

**Published:** 2023-12

**Authors:** Fergus Lewis, Ian Hall, Luca Polledri

**Affiliations:** 1 North East London NHS Foundation Trust, London, UK; 2 East London NHS Foundation Trust, London, UK

**Keywords:** Education and training, Choose Psychiatry, team-based learning, curriculum, foundation programme

## Abstract

About 45% of foundation doctors in the UK now have a placement in psychiatry. However, the current UK Foundation Programme Curriculum states that all foundation doctors need to demonstrate basic mental health-related capabilities. To address this we developed a novel teaching approach to cover these new capabilities using principles of team-based learning (TBL). This can be delivered to all foundation doctors both face to face and online using case scenarios and in no more than 4 hours over the 2-year foundation programme. The approach can be easily be replicated, but collaboration between mental health and acute trusts is essential for delivery.

The 2021 UK Foundation Programme Curriculum introduced greater emphasis on the recognition and assessment of mental disorders.^1^ It drew attention to the ‘parity of mental health’ and explicitly lists conditions that must be included in the programme (Box 1).

Box 1 Mental health conditions that foundation doctors should be able to recognise and assess^1^

Depression

Mania

Psychosis

Anxiety/panic

Delirium

Chronic cognitive impairment/dementia

Eating disorders

Substance use disorder

Somatisation disorders, including functional syndromes

It also mentions areas where foundation doctors must be able to apply knowledge of mental health legislation/treatment to patients presenting with physical health problems.^1^ This includes:

- assessing capacity and using the Mental Capacity Act 2005
- the Mental Health Act 1983
- the relevant ethical framework related to difficult decision-making
- understanding that physical disease can present with psychiatric symptoms
- serious adverse effects of common psychotropic medications
- communicating with and managing a disturbed or challenging patient and understanding risks
- explaining a diagnosis to a patient (or their carer) who has medically unexplained symptoms (MUS) or a non-organic cause for their symptoms.

It is clear from this new curriculum that mental, social and physical well-being are being prioritised together. This is very welcome as all doctors should have basic skills and confidence in this area. Our challenge was how can it be delivered to all foundation doctors making the best use of limited resources.

## Designing a teaching programme

With the publication of the new curriculum we decided to evaluate the current teaching practice in our region. The Broadening the Foundation Programme Strategy 2016–2021 has been successful at increasing foundation doctor posts within psychiatry. Since 2015, 45% of foundation doctors do at least 4 months of psychiatry.^2^ This has proved very beneficial in developing mental health skills, and is also important for inspiring the next generation of psychiatrists. This, along with the success of the Choose Psychiatry campaign, has maximised recruitment to core psychiatry training.^3^

Within the North Central and East London Foundation School, doctors who get a psychiatry rotation gain access to specific mental health teaching such as a programme of foundation-specific simulation training, involving actors as simulated patients presenting with mental health conditions listed in the new curriculum. However, doctors without a placement in psychiatry get very little specific mental health teaching, which was a gap we wanted to prioritise addressing.

## Challenges

When considering how to implement the curriculum we found a number of challenges. The topics included in the new curriculum are many and broad. We wanted to deliver a programme that did not sacrifice quality to deliver quantity but synthesised key concepts while making them clinically engaging and relevant. This is particularly important for foundation doctors who may not wish to pursue psychiatry as a career.

We were particularly concerned about the 55% of foundation doctors who do not get a psychiatry rotation. To reach these doctors we realised we would need to collaborate with the acute trusts in our region that deliver the general foundation teaching programme. The foundation programme directors had been concerned about how to deliver mental health teaching and were very happy to incorporate our teaching programme into their existing one. We also identified early on the need for collaboration between the local mental health trusts. This is particularly important as foundation doctors rotate between different hospitals and trusts in the region and we wanted to achieve consistency over the 2 years of the foundation programme and avoid repetition of material.

Another challenge we faced was thinking how to make the programme sustainable as it needed to be delivered over multiple sites. We engaged a faculty of trainers including foundation clinical supervisors in psychiatry and higher trainees in psychiatry. We emphasised the value of the training in developing mental health capabilities in all doctors, as well as encouraging recruitment into psychiatry.

## Theoretical perspective

Current research suggests that post-COVID learners are less motivated and are looking for more engaging and interactive sessions.^4^ Improved academic outcomes are directly linked to student engagement^5^ so we wanted to design a programme in which engagement was driven by students rather than the teacher. One way to do this is to adopt a more active collaborative learning among peers using few slides.^6^ This is particularly true for Kolb's theory of experiential learning.^7^ In this case we wanted to adapt the four-stage learning cycle to real-life clinical scenarios where foundation doctors could also draw on previous learning from medical school sometimes described as a ‘flipped classroom'. The key element we wanted to avoid was a passive traditional lecture style approach.

A learning method that fitted with our approach was team-based learning (TBL). This has been defined as ‘an active learning and small group instructional strategy that provides students with opportunities to apply conceptual knowledge through a sequence of activities that includes individual work, team work, and immediate feedback’.^8^ This allows for the application of small groups to large classrooms and is a collaborative active learning and teaching strategy.^9^ Learning is delivered by stimulating curiosity and discussion between trainees rather than a traditional passive approach. The model therefore does not require the facilitator to be an expert in the field. Although this can be potentially useful to address resource challenges, we found that in practice the facilitator needs a degree of expertise such as the level of a psychiatric trainee at the end of their core psychiatry training.

Many graduates of UK medical schools will have experienced similar approaches, often called case-based learning (CBL) or problem-based learning (PBL). Our approach will enable us to build on skills used in both these approaches, with more emphasis on collaborating with peers and more of a focus on the management of clinical conditions.^10^

## Method

After exploring the above we approached the acute trusts that lead the local foundation programmes. This allowed us to explore how the new curriculum could be integrated into their existing programmes, including how to monitor feedback and progress, and shared responsibilities. We then decided to develop a teaching approach using principles of TBL.

We designed ten 20–30 min case scenarios that covered the whole curriculum. The scenarios were quality assured by the other authors, and were further refined following piloting. The scenarios were designed to use no more than 4 h over the 2-year course period (Table 1). This format allows for two or three topics to be taught per hour. Some have been split into two parts to create a narrative (Box 2). This allows for more complex ideas to be introduced, such as the use of the Mental Capacity Act versus the Mental Health Act, and such a narrative approach has been shown to improve retention in learners.^11^

**Table 1:** Case studies and suggested timings

Case study	Group work	Plenary presentation	Total time
Foundation Year 1 – First 1 h session			
1. Depression and mania	Part A – 8 min	Part A – 12 min	30 min
	Part B – 4 min	Part B – 6 min	
2. Psychosis	Part A – 8 min	Part A – 12 min	30 min
	Part B – 4 min	Part B – 6 min	
Foundation Year 1 – Second 1 h session			
3. Anxiety disorders	8 min	12 min	20 min
4. Dementia	8 min	12 min	20 min
5. Delirium	Part A – 5 min	Part A – 8 min	20 min
	Part B – 3 min	Part B – 4 min	
Foundation Year 2 – Third 1 h session			
6. Eating disorders	Part A – 8 min	Part A – 10 min	30 min
	Part B – 5 min	Part B – 7 min	
7. Personality disorders and alcohol	12 min	18 min	30 min
Foundation Year 2 – Fourth 1 h session			
8. Somatisation/functional disorders	Part A – 5 min	Part A – 8 min	20 min
	Part B – 2 min	Part B – 5 min	
9. Heroin use	8 min	12 min	20 min
10. Capacity	Part A – 5 min	Part A – 8 min	20 min
	Part B – 2 min	Part B – 5 min	
Total time: 4 h over 2-year period

Box 2 Example case study: ‘Psychosis’

**Part A**

You are working in a GP practice and have been asked to conduct an urgent home visit to a 21-year-old student called Jack. His parents have reported concerns that he has stopped going outside and is not attending university. He spends most of his day isolating himself in his room and they have heard him talking to himself. He is refusing to eat or drink, reporting that his food and drink have been poisoned.

On attendance to the home you speak with Jack, who is dishevelled and very suspicious of you, accusing you of working for MI5. There is a strong smell of cannabis coming from him.

He is not aggressive. His lips look dry and he looks as if he has lost weight.

*As a group, discuss the following questions:*

1. What is your differential diagnosis and why? (Consider what other information/investigations you would want to know to narrow down your differential)
2. What risks would you need to consider?
3. What would be your management plan?

**Part B**

After involvement of the local crisis team, Jack is treated at home. Unfortunately, he jumps from the window of his two-storey house and sustains bilateral ankle fractures. He is admitted to the orthopaedic ward awaiting surgery. While on the ward he attempts to leave twice and the nurses have called you to assess him.

On the day of surgery Jack refuses to go to theatre, reporting that he is worried that MI5 will insert a chip into his ankles and claiming that he can heal himself without surgery. He would agree to a cast but the orthopaedic team believe there is significant risk of malunion and deformity and are recommending surgery.

*As a group, discuss the following question:*

1. What are your thoughts? How would you suggest to proceed?

The format of the sessions is as follows:

1. the facilitator introduces the case and questions and keeps timings; cases and explanatory slides are displayed
2. the foundation doctors discuss questions in small groups (ideally 5–8 doctors); in the case of face-to-face learning the facilitator would move around the room to help stimulate discussion and keep to task: using an online platform the trainer could enter different break-out rooms and do the same
3. the foundation doctors then bring their answers to the follow-up plenary session
4. the facilitator goes through explanatory slides covering the questions
5. the session ends with the facilitator summarising main learning points
6. two or three cases can be discussed per hour of teaching (Table 1).

We ran sessions of varying length both face to face and online. We used Likert scales to collect quantitative feedback from participants and free text to collect qualitative feedback from both participants and tutors.

This research did not require ethical approval as it is an evaluation of teaching.

## Results

We have successfully delivered the sessions both face to face and online to a total of 187 foundation doctors. We piloted both delivering three topics in 1 h during a face-to-face session in a local university hospital and six topics in a 3 h session online with a much larger cohort of doctors. Feedback from both groups showed that 97% of trainees would recommend the innovative format.

**Fig. 1:**
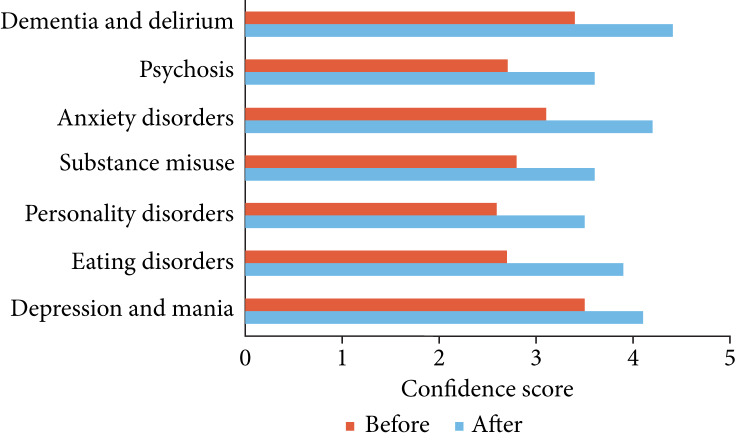
Foundation doctors’ confidence (out of 5) in the assessment and management of mental disorders before and after the virtual teaching session (independent *t*-test *t* = −4.89, two-tailed *P* = 0.0004).

Foundation doctors were asked to rate their confidence in assessment and management of mental disorders before and after the virtual teaching session. There was a significant improvement in confidence in all topic areas and mean confidence increased from 59 to 78% (Fig. 1).

Free-text comments from participants were overwhelming positively, especially regarding the interactivity of the sessions. The contrast with their previous teaching sessions was highlighted, with participants enjoying the format. Doctors particularly enjoyed peer group discussions as well as the use of breakout rooms. They enjoyed the rapid pace of the teaching and range of topics covered in the cases. Areas that needed improvement were the online session, where some doctors felt that there were too many breakout rooms, which made it repetitive. Some trainees highlighted that some people did not engage online or turn their cameras on, thus limiting their experience.

Some examples of feedback are:

‘Brilliant session and nice contrast to previous teaching’

‘The breakout rooms were a fantastic method to maximise the learning experience’

‘Clearly designed to be more engaging, really appreciate the effort. Perhaps a design with fewer moves to breakout groups would be more efficient, but thought the format worked really well’

‘I was one of the only trainees in my group with my camera on, which was a shame’.

Qualitative feedback from facilitators showed that they enjoyed the interactive style of the sessions and felt that the foundation doctors engaged enthusiastically, in contrast to other teaching. They found the resources easy to use, highly relevant to foundation training and needing very little preparation. They commented that the complexity could also be easily adjusted depending on the experience of the group. They highlighted the need for staff in the acute trusts’ medical education departments to be on hand to help with administrative work and how helpful they were. Some trainers highlighted that pastoral issues could also be picked up in face-to-face sessions and discussions about junior doctors’ well-being. A common theme was the preference of face-to-face sessions over online.

Some examples of feedback are:

‘This format is really excellent at engaging foundation doctors in discussion and making psychiatry relevant and exciting to teach!’

‘Virtual sessions longer than 2 h get quite repetitive with going in and out of breakout rooms. Face to face is definitely preferable and gives a better feel of students’ ability and mastery of the topic’.

## Discussion

### Main findings

Our data show that this teaching approach significantly increased foundation doctors’ confidence in assessment and management of mental disorders, and almost all would recommend the new format. Written feedback was very positive regarding the interactivity and structure. Although it works better face to face, it can also be delivered effectively online with breakout rooms, with fewer faculty staff. Running pilots was essential to refine the format and learn what worked well and what needed improvement.

### Learning

In line with the principles of TBL we found that sessions were most effective when facilitators prioritised time-keeping. The foundation doctors in general responded well to a fast pace. We found that a 1 h session is optimum. This can sometimes be difficult to fit into the acute trusts’ existing teaching programmes, so delivery of two 1 h sessions back to back is possible. When we piloted a 3 h session it became too repetitive and ‘Zoom fatigue’ among the foundation doctors was palpable, so we would not recommend this approach.

The complexity of material needs to be pitched at the right level. This is why the topics included in Foundation Year 2 are more complex. The facilitator should therefore be prepared to adjust the level depending on the group they are teaching, particularly as experience in mental health may be variable. Collaboration between different mental health trusts in the foundation school area is essential to prevent repetition of material when foundation doctors rotate and to ensure that the doctors experience a coherent programme over the two foundation years. Liaison with local foundation training programme directors is also essential to allow for the approach to be integrated into their existing teaching programmes in the acute trusts, but we found them very welcoming of our involvement.

As regards the online delivery of the programme, breakout rooms need to be created. The acute trusts’ medical education departments therefore need to be on hand to help deliver this.

We found that the use of breakout rooms online can also get repetitive and this is reflected in the qualitative feedback. To help alleviate this we recommend that the cases with two parts are limited to one breakout room. Discussion in the second part therefore takes place in the large group, which usually goes well as the participants are warmed up.

Other tips for facilitators include:

- moving around the room to help stimulate discussion and keep to task if teaching face to face
- entering different breakout rooms to do the same when teaching virtually; ideally another facilitator would be present to help with this
- encouraging a ‘cameras on’ approach during virtual sessions
- circulating cases to the doctors before the virtual session so they can access them in their breakout rooms
- creating a facilitator training pack setting out the structure of the programme and introducing the cases
- individual 15 min training and introductory sessions for trainers to familiarise them with the format.

### Implications and future plans

Our approach allows high-quality delivery of multiple topics using limited resources and we hope that this can be replicated in other areas of the UK. We have presented the model at the Royal College of Psychiatrists’ annual medical education conference, and as a result are facilitating its introduction in another foundation school. Further plans include developing simulation training in mental health that is accessible to all foundation doctors.

Not only is this an effective way to deliver the new curriculum – we hope that it provides the necessary momentum to challenge stereotypes and reduce stigma around mental health in the wider medical community. Our hope is that parity of esteem will remain a priority and our programme will continue to inspire the next generation of psychiatrists.

## Data Availability

The data that support the findings of this study are available from the corresponding author, F.L., upon reasonable request.

## About the authors

**Fergus Lewis** is a Fellow in Medical Education with North East London NHS Foundation Trust, London, UK. **Ian Hall** is a consultant psychiatrist and foundation tutor with East London NHS Foundation Trust, London, UK. **Luca Polledri** is a consultant psychiatrist and foundation tutor with North East London NHS Foundation Trust, London, UK.

## References

[1] Choules T, Cameron F. UK Foundation Programme Curriculum 2021. UK Foundation Programme, 2021. Available from: https://foundationprogramme.nhs.uk/curriculum/ (click on ‘The Curriculum’, then ‘UK FP Curriculum 2021 (interactive pdf)’).

[2] Royal College of Psychiatrists. Choose Psychiatry Campaign. Royal College of Psychiatrists, 2022.

[3] Royal College of Psychiatrists. RCPsych Broadening the Foundation Programme Strategy (2016–2021). Royal College of Psychiatrists, 2016.

[4] EdWeek Research Center. Data Snapshot: What Teacher and Student Morale Looks Like Right Now. Education Week, 2021 (https://www.edweek.org/leadership/data-snapshot-what-teacher-and-student-morale-looks-like-right-now/2021/01).

[5] Dyer K. Research Proof Points – Better Student Engagement Improves Student Learning. NWEA, 2015 (https://www.nwea.org/blog/2015/research-proof-points-better-student-engagement-improves-student-learning/).

[6] Toth M. Why Student Engagement is Important in a Post-COVID World – and 5 strategies to Improve It. Learning Sciences International, 2021 (https://www.learningsciences.com/blog/why-is-student-engagement-important/).

[7] Kolb D. Experiential learning: Experience as the Source of Learning and Development (Vol. 1). Prentice-Hall, 1984.

[8] Parmelee D, Michaelsen LK, Cook S, Hudes PD. Team-based learning: a practical guide: AMEE guide no 65. Med Teach 2012; 34: e275–87. 22471941 10.3109/0142159X.2012.651179

[9] Burgess A, van Diggele C, Roberts C, Mellis C. Team-based learning: design, facilitation and participation. BMC Med Educ 2020; 20(suppl 2): 461. 33272267 10.1186/s12909-020-02287-y PMC7712595

[10] McLean SF. Case-based learning and its application in medical and health-care fields: a review of worldwide literature. J Med Educ Curric Dev 2016; 3: JMECD.S20377. Available from: 10.4137/JMECD.S20377 29349306 PMC5736264

[11] Easton G. How medical teachers use narratives in lectures: a qualitative study. BMC Med Educ 2016; 16: 3. 26742778 10.1186/s12909-015-0498-8 PMC4705637

